# Views of senior health personnel about quality of emergency obstetric care: A qualitative study in Nigeria

**DOI:** 10.1371/journal.pone.0173414

**Published:** 2017-03-27

**Authors:** Friday Okonofua, Abdullahi Randawa, Rosemary Ogu, Kingsley Agholor, Ola Okike, Rukayat Adeola Abdus-salam, Mohammed Gana, Eghe Abe, Adetoye Durodola, Hadiza Galadanci, WHARC WHO FMOH MNCH Implementation Research Study Team

**Affiliations:** 1 Women’s Health and Action Research Centre, Benin City, Edo State, Nigeria; 2 University of Medical Sciences, Ondo City, Ondo State, Nigeria; 3 Ahmadu Bello University, Zaria, Kaduna State, Nigeria; 4 University of Port Harcourt, Port Harcourt, Rivers State, Nigeria; 5 Central Hospital, Warri, Delta State, Nigeria; 6 Karshi General Hospital, Federal Capital Territory, Abuja, Nigeria; 7 Adeoyo Hospital, Ibadan, Oyo State, Nigeria; 8 General Hospital, Minna, Niger State, Nigeria; 9 Central Hospital, Benin City, Edo State, Nigeria; 10 General Hospital, Ijaye, Abeokuta, Ogun State, Nigeria; 11 Aminu Kano Teaching Hospital, Kano State, Nigeria; Centre for Injury Prevention and Research, Bangladesh (CIPRB) & Örebro University, Sweden, BANGLADESH

## Abstract

**Background:**

Late arrival in hospital by women experiencing pregnancy complications is an important background factor leading to maternal mortality in Nigeria. The use of effective and timely emergency obstetric care determines whether women survive or die, or become near-miss cases. Healthcare managers have the responsibility to deploy resources for implementing emergency obstetric care.

**Objectives:**

To determine the nature of institutional policies and frameworks for managing obstetric complications and reducing maternal deaths in Nigeria.

**Methods:**

Thirty-six hospital managers, heads of obstetrics department and senior midwives were interviewed about hospital infrastructure, resources, policies and processes relating to emergency obstetric care, whilst allowing informants to discuss their thoughts and feelings. The interviews were audiotaped, transcribed and analyzed using Atlas ti 6.2software.

**Results:**

Hospital managers are aware of the seriousness of maternal mortality and the steps to improve maternal healthcare. Many reported the lack of policies and specific action-plans for maternal mortality prevention, and many did not purposely disburse budgets or resources to address the problem. Although some reported that maternal/perinatal audit take place in their hospitals, there was no substantive evidence and no records of maternal/perinatal audits were made available. Respondents decried the lack of appropriate data collection system in the hospitals for accurate monitoring of maternal mortality and identification of appropriate remediating actions.

**Conclusion:**

Healthcare managers are handicapped to properly manage the healthcare system for maternal mortality prevention. Relevant training of healthcare managers would be crucial to enable the development of strategic implementation plans for the prevention of maternal mortality.

## Introduction

Nigeria is one country in sub-Saharan Africa that did not achieve the Millennium Development Goal (MDG) 5 aimed at reducing the rate of maternal mortality by 75% between 1990 and 2015. The country now accounts for about 14% of global maternal deaths and is second to India in terms of the absolute numbers of maternal deaths globally. Of the many factors that account for high rates of maternal mortality in Nigeria, poor access to emergency obstetric services is one of the most important factors that can be addressed immediately to reverse the trend.

Several reports indicate that the late arrival in hospital by women experiencing pregnancy complications is one of the most important background factors that lead to maternal mortality in Nigeria [1, 2, 3]. However, when women arrive late in hospital, the use of efficient, effective and timely emergency obstetric care in referral hospitals often determines whether women would live or die, or end up as near-miss cases. Available evidence suggests that in many secondary and tertiary care hospitals in Nigeria, the quality of emergency obstetric care is often sub-optimal [4, 5, 6], and is not appropriately designed to respond to women experiencing exacerbated pregnancy complications. This is evidenced by studies that report higher case fatality rates for women arriving in Nigerian secondary and tertiary hospitals with various obstetric complications [7, 8, 9] as compared to rates reported from more developed parts of the world. Oladapo et al [10] in a recently published study reviewing 998 maternal deaths and 1451 near-miss cases in Nigeria emphasized the point that getting to maternity care centres is not enough: there must be a purposefully designed action plan and effective services to prevent maternal deaths and severe maternal morbidity.

Health care managers and administrators have the responsibility to deploy resources for implementing emergency obstetric care that can help manage the complications that lead to maternal deaths in a prompt and effective manner. We hypothesize that if hospital managers and senior maternal health personnel develop strategic policies towards the implementation of priority policies and programs in emergency obstetric care, it will result in substantial improvement in the quality of emergency obstetric services and therefore the reduction in maternal mortality in Nigeria. To date, there has been no empirical information on what hospital managers and senior maternity health personnel do or do not do with respect to generating commitment for providing emergency obstetric services in the country.

The objective of this paper is to determine what hospital managers and decision-makers in selected secondary and tertiary hospitals in Nigeria know about the seriousness of maternal mortality in their hospitals and to identify the nature of the policies and programs they put in place to deal with the problem. We believe this paper will stimulate ideas on ways to improve policies and programs for providing optimal emergency obstetrics services in referral hospitals across the country.

## Methodology

Nigeria’s health care system is founded on a three-tier system–primary, secondary and tertiary care. Primary health care provides preventive and referral maternity services, while emergency obstetric care (EMOC) services are offered in secondary and tertiary care facilities. In actuality, secondary care facilities (General Hospitals) are more accessible and attend to a greater number of obstetric emergencies as compared to tertiary care facilities. We designed to be a nationally representative study to enable generalizability of the findings to the larger Nigerian context. Thus we 8 sampled hospitals from 4 out of the six geo-political zones of the country (two each per geo-political zone). There are at least 6 States by zone in Nigeria–we choose two States from each of the 4 zones. The hospitals consisted of 6 secondary hospitals (2 in northern states and 4 in southern states) were secondary hospitals; while only 2 (both from northern Nigeria) were tertiary/teaching hospitals.

**Fig 1:**
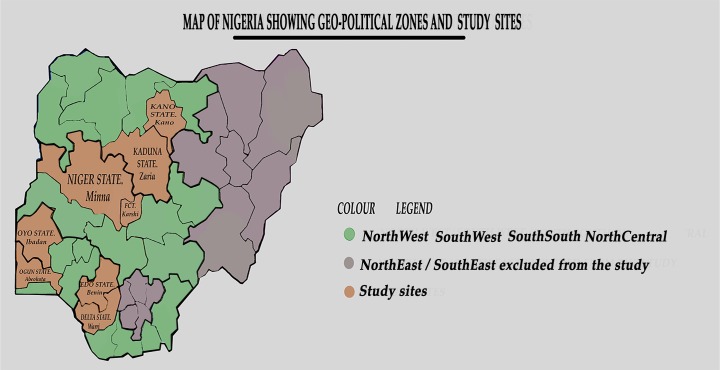
Map of Nigeria showing the geopolitical zones, states and study sites.

The two teaching hospitals selected for the study were the Aminu Kano Teaching Hospital in Kano, Kano State and the Ahmadu Bello University Teaching Hospital, Kaduna in Kaduna State. The secondary care facilities were: Adeoyo Maternity Hospital, Ibadan, Oyo State; General Hospital, Ijaye, Abeokuta; General Hospital, Minna, Niger State; General Hospital, FCT; General Hospital, Warri, Delta State; and General Hospital, Benin City, Edo State. Each has a mission to deliver emergency obstetric services to pregnant women and attend to not less than 2000 pregnant women each year. We paired hospitals in neighboring States, so as to provide opportunity for comparison of results within and between States and zones. The States and sites of the chosen hospitals are presented in Fig 1.

We conducted Key Informants in-depth interviews (IDI) with hospital managers and senior maternity health personnel in each of the in 8 secondary and tertiary care hospitals. Five interviews were conducted in each hospital, except the Central Hospital, Warri and the Aminu Kano, Hospital, Kano where four and three interviews were conducted respectively. Thus, a total of 36 interviews with key policy makers were conducted across the 8 hospitals. This consisted of interviews with Chief Medical Directors, Program Managers, Heads of Departments of Obstetrics and Gynaecology and Paediatrics, and Senior Nurses and midwives.

Each interview consisted of questions on the overall policies in the hospital especially those that relate to maternal health, the priority accorded to maternal health in the disbursement and allocation of resources, perceptions about maternal morbidity and mortality and whether any specific policies and action plans are in place to prevent maternal and perinatal deaths. See interview guide S1 File in supporting information. The interviews were semi-structured, enabling interviewers to collect information about hospital infrastructure, resources, policies and processes relating to emergency obstetric care for major complications, whilst still giving informants the opportunity to report on their own thoughts and feelings. The interviews were audio-taped and transcribed in each hospital and the transcripts were later forwarded to the coordinating office.

Qualitative data analysis package Atlas ti 6.2 was used for coding and for data analysis. At the first step, transcripts were assigned into Atlas ti and open coding was used to generate themes following the guide used for the study, and emerging concepts. At the second level, the codes were organized into analytical categories in form of code families in relation to the study objectives. Further analysis consisted of description of the content and form of transcripts conducted in each site, followed by a review and comparison of the results between the sites. The results enabled us to gain insights into the nature of the policies in each site as well as women’s perceptions regarding the quality and effectiveness of services.

Ethical approval for the study was obtained from the World Health Organization and the National Health Research Ethics Committee (NHREC) of Nigeria–number NHREC/01/01/2007–16/07/2014, renewed in 2015 with NHREC 01/01/20047-12/12/2015b. The participants gave written informed consent.

## Results

We first posed the question as to how hospital managers perceive the seriousness of maternal mortality as a major challenge in their hospitals. A significant majority of respondents (20/36) reported that maternal mortality is a serious complication of pregnancy in all the hospitals. Such answers were particularly frequent at the Central Hospital, Benin City, General Hospital, Abeokuta, Aminu Kano Hospital, Kano, ABU hospital, Kaduna and the General Hospital, Minna. Some respondents tried to quantify the extent of the problem in various ways, but the reports were not internally consistent. Responses such as “25% of the patients managed here experience these complications” (a respondent from Oyo State); “less than 10% of patients coming to this hospital have these complications” (another respondent from Oyo State); and “2% of patients coming to this hospital have these cases” (a third respondent from Oyo State) testify to the lack of unanimity in the quantification of the nature of the problem in each hospital.

Respondents also gave various insights into the nature of the problem in the hospitals. Among those who perceive maternal mortality to be a serious problem, the majority reported that the problem was more common in un-booked women (women who did not originally initiate care in the hospitals) as compared to booked women. Other reasons proffered by the respondents for high rates of maternal mortality in the hospitals included: shortage of skilled health personnel, including consultant physicians and anesthetists (Ogun State), systemic failure (Ogun State), women preferring to deliver in Churches and traditional homes due to financial constraints (Ogun State, Oyo State), post-caesarean complications (Adeoyo Hospital), lack of life saving drugs (Central Hospital, Benin City), and late arrival of patients in hospital (Benin City).

Respondents from Central Hospital, Warri, the Adeoyo hospital, Ibadan and the General Hospital Minna were more likely to report that maternal mortality is either not serious or is low. In Warri, one respondent answered thus “In Central Hospital, these problems are a little bit reduced compared to what is happening in other hospitals because we have capable doctors”. Another respondent from the same hospital said “Maternal mortality in this facility is relatively low because of the free maternal care. Money has been a deterrent before now but presently there is no charge–so, most women show up early to access care”. A respondent from Adeoyo hospital said “the problem has reduced. There has been no mortality in this hospital since the past few months”. However, no reasons were adduced for this reduced rate of maternal mortality in the hospital.

### Medical and social causes of maternal mortality

We asked respondents to list the most common obstetric causes of maternal mortality in their health facilities. Eclampsia and the complications of pregnancy induced hypertension was the most frequently mentioned attributable cause, reported by 23/36 respondents. All respondents in Central Hospital, Benin city reported eclampsia as the most common cause of maternal mortality, while four out of five respondents also reported eclampsia in ABUTH Kaduna and Adeoyo Hospital, Ibadan. PPH was the second most common cause of maternal mortality listed by all respondents, followed by obstructed labour and antepartum haemorrhage (APH). APH was listed only by Central Hospital, Warri as an important cause of maternal mortality. Other causes of maternal mortality mentioned in the different hospitals included maternal anaemia (Adeoyo hospital), ruptured uterus (Abeokuta and Adeoyo Hospital), HIV encephalopathy (Central Hospital, Warri only), haemoglobinopathy (Abeokuta), maternal infection (ABUTH, Kaduna, Adeoyo Hospital and Aminu Kano Hospital, Kano), post-abortal sepsis (Abeokuta, Ogun State).

When asked about the socioeconomic causes of maternal mortality, nearly all respondents from all health facilities reported the delayed presentation of patients in hospital. Patients with complications either come late from their homes or from traditional or religious places of delivery. Some come referred late from private health facilities. A respondent from Central Hospital, Benin replied as follows: “Unbooked cases referred from other hospitals (private) are a major problem. Before we manage (them), they come too late”. Also, the lack of adequate skilled personnel to manage complications was mentioned as a key factor at the General Hospital in Niger State and in Ogun State.

We particularly wanted to know how service providers ranked patient factors versus health facilities-related factors in their attribution of socio-economic causes of maternal mortality. It was of interest that the large majority reported factors related to the patients or their families as key in predisposing to maternal mortality rather than those related to inadequacies in the health care system. The most important factor that featured in nearly all the responses was poverty/financial constraint/unemployment. These were reported by all respondents in Adeoyo Hospital, Ibadan, and the General Hospital, Minna, Niger State, and by most of the respondents in Kaduna, Abeokuta, and Benin City. On poverty, a respondent in Benin City responded as follows: “Lateness of the patient to hospital as a result of poverty is a problem; they may be willing to come but money will delay them and you find that they come late and before intervention, and because it is too late to follow up, the patient ends up dying”. Another respondent also from Benin City, answered as follows: “What I can say right now is the nature of the economic downturn in our country and our State. These pregnant women don’t eat what they are supposed to eat, they are not cared for well…. Because the finances are not there, they cannot make payment for prescribed drugs”. Also another said “the delay is there because what is really responsible is the money aspect. Due to lack of money, they prefer to go to quacks because that would be easier for them as they are not charged”.

Other key answers that featured prominently in the question on socio-economic determinants of maternal mortality were “ignorance”, “lack of women’s education/illiteracy”, “preference for traditional birth attendants” “cultural preference for vaginal delivery” and “poor role of husbands and families”. Less prominent were answers such as “lack of antenatal care”, “religious doctrines that prevent women from using evidence-based care”, “young age of patients”, “negative peer influences”, “long distances”, and “sex preferences”. Some quotes that illustrate these responses include: A respondent from Oyo posited “Culture has a role to play but it is on the decline. “Culture plays a role in wanting vaginal delivery and declining caesarean section”. Another respondent from Oyo said “Age–adolescent pregnancies, marital characteristics of the adolescent versus unmarried adolescent. The unmarried adolescent is at risk of complications, lack of social support also contributes to this”. A respondent from Warri, Delta state mentioned the negative effects of some religious practices as follows: “Most patients come from God’s Grace Ministries–unfortunately most grand multips come from this Church and family planning is forbidden for them”.

Among respondents who blamed health services for maternal mortality, most mentioned “type 3 delay”, “delayed referral” and “mismanagement of complications” as important factors. Such reports came mainly from respondents in Oyo and Abeokuta (Ogun State), while ABUTH, Kaduna and Central Hospital, Benin mentioned the high cost of fees charged in hospital as disincentives to patients using evidence-based care.

### Existing policies for preventing maternal and perinatal deaths

When asked whether there are existing policies on prevention of maternal and perinatal deaths in the hospitals, several respondents from Central Hospital, Warri, Central Hospital, Benin City and the Aminu Kano Hospital, Kano unanimously replied that they were not aware of such policies. However, although respondents from Kaduna, Niger, Ogun and Oyo replied that the hospitals had policies, none could provide written or documentary evidence of the policies. It would appear that the hospitals had protocols and guidelines, but not strategic policies purposely designed and known by staff for preventing maternal and perinatal policies. Common responses to this question in these hospitals were: “1) Yes, provision of service before payment, provision of blood, use of misoprostol in management of PPH–Niger State; 2) Yes, there are treatment guidelines and protocols are periodically circulated–Respondent from Abeokuta, Ogun State; 3) Yes, we have developed some protocols in pre- eclampsia-eclampsia, and in postpartum haemorrhage in accordance with national guidelines. We also have monthly maternal mortality meetings and we just developed perinatal mortality meeting with paediatrics which we do monthly and we are able to find the causes of mortality and we are able to prevent any occurrences”.

Thus is all hospitals studied, it was evident that efforts have not been made by hospital Management to develop and document policies and action plans for preventing maternal deaths.

We then asked whether the hospitals conduct maternal and perinatal audits either separately or combined that will enable the identification and correction of errors attended with management of complications that lead to maternal and perinatal deaths. Nearly all reported that they have maternal audits, except Central Hospital, Benin City where all respondents reported that no such audits existed. The Central Hospital, Warri reported that they conduct daily morning reviews through which they discuss maternal deaths. The frequency of such meetings ranged from daily (Central Hospital, Warri) to weekly (ABUTH, Kaduna) to monthly (Teaching Hospital, Kano; General Hospital, Minna; State Hospital, Abeokuta; and Adeoyo Hospital, Oyo). Examples of the answers given include: 1) Yes, monthly. We have allocated a consultant to be in charge of maternal deaths, so any maternal death is reported to this consultant (name given), for record purpose and at the end of each month, there will be a detailed report on that and the aim is to find out why the death occurred so as to prevent future occurrence and not to apportion blame (Kano respondent)”; 2) Yes, through monthly collection and review in a monthly meeting (Minna, Niger); 3) Mortality review meetings–presentation of mortality to identify main cause of death, remote cause of death, and contributory cause of death, and how the patient could have been helped (Adeoyo Hospital, Oyo)”. However, none of the hospitals could show a report of any of the meetings, and action plans taken to correct deficiencies. Also, the Federal Ministry of Health had issued guidelines and systematic steps for conducting maternal death reviews and surveillance. From the interviews conducted, there was no evidence that any of the hospitals was using these guidelines and steps to conduct maternal death reviews. Only the ABUTH in Kaduna reported that they also conduct Perinatal Death Audits, but again the records were not shown. One respondent from this hospital explained as follows: “Any time there is a perinatal death, we summon the team to a meeting with a view to presenting the case. At the meeting, we look at the nature and type of intervention given and the logistics governing the intervention. We examine the errors in the process of diagnosis and management and suggest appropriate measures for intervention. Lastly, we seek to address the issue with the help of hospital management”. And none of the hospital reported that they conducted combined Maternal and Perinatal Death Reviews.

Emergency Obstetrics Management of Eclampsia, PPH and Obstructed Labor We also investigated the use of standard protocols and guidelines known by all obstetric staff for the management of PPH, Eclampsia and Obstructed labour in the hospitals. Respondents at the Central Hospital, Warri, Central Hospital, Benin, General Hospital, Niger State and State Hospital, Abeokuta generally reported that they did not have such protocols. By contrast, respondents from Aminu Kano Hospital, and Adeoyo Hospital, Oyo indicated that they had standard protocols for the management of major obstetrics complications. Respondents from the ABUTH, Kaduna gave divergent opinion on this. While two respondents reported that no such protocols existed, two others reported that the hospital has protocol for the management of the complications.

In all, none of the hospitals could show copies of documented protocols for the management of these complications. The common responses to the question included narratives of what health providers normally do when patients with these complications present as emergencies. Some of these narratives tallied with internationally and nationally accepted guidelines, while others appeared to be generic for the individual hospitals. For example, some of the responses of the management of PPH included “ABC of life”, “blood transfusion”, “bladder catheterization”, “intravenous infusion”, and “internal artery ligation”, but there were no documents were the stepwise use of these interventions were reported.

As for eclampsia, respondents reported the use of “stabilization of the patient”, “control of blood pressure”, “administration of magnesium sulphate”, “surgical intervention when necessary” and “adequate nursing care”. Again none of these processes appear to have been documented in any of the hospitals.

Also, respondents reported “emergency caesarean section”, “bladder catheterization”, “destructive operations”, “administration of antibiotics” as standard procedures for managing obstructed labor with no evidence that any of these procedures were documented in the hospitals. For all the complications, there was no report of health care workers being trained or updated on methods to use the most recent treatment methods and no charts or reminders were reported to be in place in the maternity sections for workers to use the new methods.

### Views on quality of emergency of obstetrics care

We asked the respondent to rate the quality of Emergency Obstetrics care in their hospitals for the treatment of PPH, eclampsia, and obstructed labor on a scale of 1 to 5–1 (one) being the lowest, and five (5) the highest. They were requested to rate with respect to quality of hospital facilities for the management of these complications, the use and adherence to standard treatment protocols, the training and competency levels of staff and the availability of audit, reflection and improvement opportunities for the complications.

The results are presented in Table 1. As shown, when taken in totality, the five hospitals scored just above averages in all the domains studied. But when analyzed by individual hospitals, the Central Hospital, Warri scored low in availability of protocols and hospital facilities, but scored relatively high in staff training and audit/reflections. The Central Hospital, Benin scored low in protocols and hospital facilities, was average in audit/reflections, but was relatively appraised higher by staff on staff training/competence. The Adeoyo Hospital, Ibadan was scored very high on protocol availability, but only scored averagely on the other 3 domains. By contrast, the Hospital in Abeokuta was scored low in protocol availability but above average in the remaining three areas. Interesting, the Aminu Kano Hospital in Kano was scored high by staff in all four domains. The ABU Teaching Hospital was rated below average in protocol availability but rated well above average in the remaining three areas. The Hospital in Minna slightly above average in staff training and competence and well above average in the other three areas.

**Table 1:** Rating of aspects of quality of emergency obstetrics care by the Key Informants in the Hospitals (lowest rating = 1, highest = 5 per category).

	Warri	Benin	Ibadan	Abeokuta	Kano	Kaduna	Minna	Total
No of Respondents	3	5	5	5	3	5	5	31
Max expected scores	15	25	25	25	15	25	25	145
Availability of protocols and policies	3, 1, 1 (5)	2, 1, 1 (4)	5, 5,4,5, 4 (23)	3, 1, 1, 3, 2 (10)	5, 1, 5 (11)	3,3, 3, 3, 3 (12)	4, 2, 3, 2, 3, 2 (16)	83
Staff Training & Competence	1, 4, 4 (9)	3, 4, 3 (10)	3, 3, 3, 4 (13)	3, 3, 3, 3, 3 (15)	4, 2, 5 (11)	4, 2, 3, 3, 4 (16)	5, 3, 1, 2, 3 (14)	88
Audit, reflection and improvement opportunities	5, 4, 2 (11)	1, 3, 4 (8)	3, 3, 2, 2, 3 (13)	3, 3, 3, 3, 3 (15)	5, 4, 4 (13)	3,4,3, 2, 3(15)	3, 1, 5, 5, 3 (17)	92
Hospital facilities	3, 3, 1 (7)	1, 1, 2 (4)	3, 3, 4, 2, 2 (14)	2, 3, 5, 3, 3(16)	4, 5, 4 (13)	3,3, 5, 3, 2 (16)	3, 4, 4, 3, 4 (18)	88

### Views on methods to improve emergency obstetrics care

Key informants were then asked to make recommendations on ways to improve the quality of emergency obstetrics care in their various hospitals. The results listing all recommended improvements are shown in Table 2. Of all the recommendations, personnel training and re- training, recruitment and improved remuneration was the most common recommendation that featured prominently in Warri, Ibadan and Abeokuta. This was followed by recommendation on replacement of obsolete equipment/provision of essential services, e.g. caesarean sections and provision of drugs (especially in Ibadan, Abeokuta and Minna). Other recommendations that were high on the list included health talks to patients, the development of protocols to which staff should be adherent, improved funding of maternal health and improved blood bank and laboratory facilities.

**Table 2:** Aspects of emergency obstetrics care to be improved–recommendations by key informants.

	Warri	Benin	Ibadan	Abeokuta	Kano	Kaduna	Minna	Total
No of Respondents								
**Key Recommendations**								
Protocols and policies to be in place–improved standard of care	1	-	1	-	3	-	-	5
Replacement of obsolete equipment/provision of essential services, e.g. c/s and drugs	1	-	5	2	-	1	2	11
Improved funding of maternity services/indigent fund	1	-`	1	-	2	-	-	4
Health talks to patients–including pre-conceptional care	1	2	2	-	-	-	2	7
Improved transportation to enable women reach hospitals in time	-	-	-	-	-	1	-	1
Reduction of hospital costs for patients	-	-	-	-	-	1	-	1
Personnel training and re- training, recruitment & improved remuneration	3	1	5	4	1	1	2	17
Improvement of anesthetic services	-	-	1	-	-	-	-	1
Provision of regular water and electricity	-	-	2	-	-	-	-	2
Improved blood bank services	-	-	2	1	-	-	-	3
Better coordination of multi- disciplinary clinical management	-	-	1	-	-	-	-	1
Proper record keeping–improved records	-	-	-	-	1	-	-1	
Improved laboratory services	-	-	-	3	-	-	-	3
Need to improve quality of maternal health care	1	-	-	-	-	-	-	1
Spousal involvement in antenatal & delivery care	-	-	1	-	-	-	-	1
Interpreter to bridge language barrier	-	-	1	-	-	-	-	1

## Discussion

The objective of this qualitative study was to investigate the nature of maternal and perinatal services in secondary and tertiary hospitals in Nigeria as reported by health care managers managing the institutions. To the best of our knowledge, this is the first such study in Nigeria, which was designed to help determine how health administrators think about the services they supervise, and what recommendations they make for improving the quality of maternal and perinatal services and reducing maternal and perinatal deaths. The results showed that although senior health providers and managers in the seven hospitals studied are generally knowledgeable about maternal health, they did not appear to prioritize it in decision-making. There was substantial variability in the way the managers of hospitals identified the seriousness of the problem of maternal mortality. Many did not identify maternal or perinatal mortality as important indicators for measuring the success of hospitals, and many could not provide actual verifiable figures on the extent of maternal mortality in their hospitals. Appropriate data were shockingly lacking in all the hospitals visited. Due to the currency of the debate surrounding the need to reduce maternal and perinatal mortality in Nigeria, it was expected that top managers of secondary and tertiary hospitals would have readily available summary data on the extent of maternal mortality in their institutions but to our surprise, none of the hospitals could provide such information.

Many hospitals did not have specific policies and action plans for preventing maternal deaths and managing complications that lead to deaths. Some of the answers to questions relating to policies appear to be those known to individual staff, but there did not appear to be definitive institutional policies and action plans developed and known by all staff for managing complications and reducing maternal deaths.

Although some of the hospitals reported that they had standard treatment protocols, none could show an example of a protocol or policy that is taught or disseminated to all staff. Without standard protocols or policies, staff in these institutions would depend on individual knowledge and preferences which may sometimes be conflicting. Similarly, although the Federal Ministry of Health has recommended the use of maternal and perinatal death reviews and surveillance [11] as a way to help institutions managing obstetrics complications to identify deficiencies that lead to maternal mortality so as to carry out remediating actions, there was no evidence in any of the hospitals that this system has been put in place. The FMOH has also recommended standard treatment protocols for the management of obstetric emergencies [12] and also standard protocols and guidelines for the implementation of Maternal and Perinatal Death Reviews and Surveillance[11]. Although some of the health institutions reported that they did daily, weekly or monthly maternal death reviews respectively, none followed the standard guidelines prescribed by the FMOH. Many of the health institutions visited did not appear to do perinatal reviews periodically; many did not integrate perinatal reviews into maternal death reviews; and only one hospital reported that they occasionally carry out perinatal reviews separately.

Clearly, there is a need to develop a mechanism for encouraging Clinical Managers in health care institutions that manage severe obstetrics to prioritize the provision of quality and essential services. Such a system should consist of capacity building and re-training opportunities for senior managers to enable them understand the background causes of maternal and perinatal deaths and to adopt strategic approaches (including appropriate policy development) in their health institutions for combating them.

The assessments provided by senior health providers indicate that deficiencies related to health workers, availability of essential services, poor funding, organizational factors and delays were associated with poor quality of obstetrics care in the health system. Similar systems delay and difficulties have been reported in many reviews of the quality of maternal and perinatal care provided in several hospitals in Nigeria [13, 14, 15]. It is noteworthy that despite their being in positions to tackle these deficiencies, the health care managers did not appear to have the full agency to deal with the situation. This suggests that a comprehensive turn-around of the system including focusing on accountability and funding instruments would be needed to reverse the trend.

The key recommendations for improving emergency obstetrics care services made by the health managers also tallied with their assessment of the nature of the deficiencies. These include: personnel training and re-training, recruitment and improved remuneration for staff, replacement of obsolete equipment/provision of essential services, provision of essential drugs, health talks to patients, the development of protocols to which staff should be adherent, improved funding of maternal health and improved blood bank and laboratory facilities. As many of these recommendations were specific to the health facilities, it is possible to apply specific remediating actions to the institutions studied.

In conclusion, the results of this study indicate that health care managers are able to provide reliable information on the nature and quality of maternal health services in their institutions. The results have implications for the design of programs for improving the quality of emergency obstetrics services in secondary and tertiary health care institutions in Nigeria. We believe that substantial gains can be made if efforts are focused on institutional policy development, staff training and re-training, the development and uptake of standard practice guidelines, patients’ education, costs alleviation for patients seeking care, and increased funding of maternal health services.

## Supporting information

S1 File Interview guide. (DOC)
