# Supplementary material for: Views of senior health personnel about quality of emergency obstetric care: A qualitative study in Nigeria
Source: PLoS One. 2017 Mar 27;12(3):e0173414. doi: 10.1371/journal.pone.0173414 (PMC5367679; doi:10.1371/journal.pone.0173414)
Supplement: S1 File — (DOC) [file pone.0173414.s001.doc]

**Key informant Interview Guide**

*(For program managers, hospital administrators, heads of O&G Departments and Midwifery staff in charge of Antenatal and Postnatal Clinics and labor wards: to be completed at Phase 1, needs assessment study)*

1. Hospitals often experience the problem with women who die during pregnancy (maternal mortality). How serious is the problem in this hospital? (To probe for perceptions on level of severity)
2. What are the leading clinical causes of maternal deaths in the hospital? Please, rank them in the order of their frequency in the hospital.
3. In your experience, what social factors apart from clinical factors are responsible for these deaths? (Probe for types of delays, patient’s characteristics, etc.)
4. Does this hospital have specific policies and programs for dealing with the problem? If so, list them and if possible show documented policies and programs
5. Does the hospital carry out maternal and perinatal audit? If so, please describe the method, and show evidence (if documented)
6. Can you tell me about hospital policy in relation to care and management of patients with emergency PPH, PE & OL (describe each in turn)
7. Please describe the typical processes that would be followed in managing patients with each of these conditions
8. In your opinion, do all clinicians and midwives operate in the same way?
9. Are there aspects of care that you feel could be improved and if so, what?
10. On a scale of 1-5 (with five being best), how would you rate:

- Hospital facilities (in relation to emergency obs patients with the 3 conditions)
- Availability of policies and protocols
- Use of and adherence to protocols
- Training and competency levels of staff
- Audit, reflection and improvement opportunities
